# A New Approach to Postoperative Rehabilitation following Mosaicplasty and Bone Marrow Aspiration Concentrate (BMAC) Augmentation

**DOI:** 10.3390/biomedicines12061164

**Published:** 2024-05-24

**Authors:** Robert Gherghel, Ilie Onu, Daniel Andrei Iordan, Bogdan Alexandru Antohe, Ioana-Irina Rezus, Ovidiu Alexa, Luana Andreea Macovei, Elena Rezus

**Affiliations:** 1 Department of Orthopedics and Trauma Surgery, Piatra Neamt Emergency Hospital, 700115 Piatra Neamt, Romania; gherghelrobert@yahoo.com; 2 Departments of Orthopedy and Physiotherapy, Medlife-Micromedica Clinic, 610119 Piatra Neamt, Romania; 3 Department of Biomedical Sciences, Faculty of Medical Bioengineering, University of Medicine and Pharmacy “Grigore T. Popa” Iasi, 700454 Iasi, Romania; 4 Center of Physical Therapy and Rehabilitation, “Dunărea de Jos” University of Galati, 800008 Galati, Romania; daniel.iordan@ugal.ro; 5 Department of Individual Sports and Kinetotherapy, Faculty of Physical Education and Sport, “Dunarea de Jos” University of Galati, 800008 Galati, Romania; 6 Department of Physical and Occupational Therapy, “Vasile Alecsandri” University of Bacau, 600115 Bacau, Romania; antohe.bogdan@ub.ro; 7 Department of Dermatology, Faculty of Medicine, “Grigore T. Popa” University of Medicine and Pharmacy Iasi, 700115 Iasi, Romania; ioanairinarezus@yahoo.co.uk; 8 Department of Orthopaedic and Traumatology, Faculty of Medicine, “Grigore T. Popa” University of Medicine and Pharmacy of Iasi, 700115 Iasi, Romania; ovidiu.alexa@umfiasi.ro; 9 Department of Rheumatology and Rehabilitation, Faculty of Medicine, “Grigore T. Popa” University of Medicine and Pharmacy Iasi, 700115 Iasi, Romania; elena.rezus@umfiasi.ro

**Keywords:** chondral defects, mosaicplasty, bone marrow aspirate concentrate, rehabilitation, cartilage repair

## Abstract

Background: Chondral defects in the knee present a significant challenge due to their limited self-healing capacity, often leading to joint degeneration and functional disability. Current treatments, including surgical approaches like mosaicplasty and regenerative therapies such as bone marrow aspirate concentrate (BMAC) augmentation, aim to address these defects and improve patient outcomes. Materials and Methods: This study conducted a single-center, randomized controlled trial to evaluate the efficacy of different treatment approaches and rehabilitation protocols for chondral defects. Thirty-seven subjects presenting with symptomatic chondral or osteochondral defects (>3 cm^2^) in the weight-bearing region of the femoral condyle were partitioned into three groups, and underwent mosaicplasty with or without BMAC augmentation, followed by either a 6-week or 12-week rehabilitation program. Group 1 (*n* = 10) received mosaicplasty combined with BMAC augmentation and engaged in a twelve-week two-phase rehabilitation protocol. Group 2 (*n* = 15) underwent mosaicplasty alone and participated in the same twelve-week two-phase rehabilitation regimen. Meanwhile, Group 3 (*n* = 12) underwent mosaicplasty and underwent a shorter six-week one-phase rehabilitation program. Clinical assessments were performed using the visual analog scale (VAS) for pain, goniometry for the knee’s range of motion (ROM), manual muscle testing (MMT) for quadricep strength, and the Western Ontario and McMaster University Arthritis Index (WOMAC) for functional evaluation in three test phases. Results: Significant differences in WOMAC scale scores were observed between the three groups at the intermediate (F(2, 34) = 5.24, *p* < 0.010) and final (F(2, 34) = 111, *p* < 0.000) stages, with post hoc Tukey tests revealing variations shared among all three groups. The between-group analysis of the VAS scale demonstrated no statistically significant difference initially (F(2, 34) = 0.18, *p* < 0.982), but significant differences emerged following the intermediate (F(2, 34) = 11.40, *p* < 0.000) and final assessments (F(2, 34) = 59.87, *p* < 0.000), with post hoc Tukey tests revealing specific group variations, notably between Group 1 and both Group 2 and Group 3, and also between Group 3 and Group 2. The between-group analysis of quadricep muscle strength using MMT scores revealed no statistically significant differences initially (F(2, 34) = 0.376, *p* < 0.689) or following the intermediate assessment (F(2, 34) = 2.090, *p* < 0.139). The one-way ANOVA analysis showed no significant difference in the knee ROM initially (F(2, 34) = 1.037, *p* < 0.366), but significant differences emerged following intermediate (F(2, 34) = 9.38, *p* < 0.001) and final assessments (F(2, 34) = 11.60, *p* < 0.000). Post hoc Tukey tests revealed significant differences between Groups 1 and 2, Groups 1 and 3, and Groups 2 and 3 at intermediate and final assessments. Conclusions: The patients who received BMAC augmentation and completed a 12-week rehabilitation protocol had significantly better outcomes in pain relief, knee function, and ROM when compared to those who did not receive BMAC augmentation or those who completed a shorter rehabilitation period. Our findings suggest that combining mosaicplasty with BMAC augmentation and a comprehensive rehabilitation program can lead to superior clinical outcomes for patients with chondral defects in the knee.

## 1. Introduction

Untreated chondral defects of the knee can lead to joint degeneration and disability in joint function [1]. Given its minimal ability to self-heal, even in small focal lesions, it can become the cause of severe osteoarthritis. Once the articular cartilage has been damaged, the chances of spontaneous recovery are low, and current technology is not sufficiently efficient to repair the lesion [2]. Chondral defects are being addressed surgically, non-surgically, and through cartilage regenerative medicine using tissue engineering. 

Technological advances and research in tissue and stem cell engineering have shown promising results, especially in the direction of mesenchymal stem cells [3]. Some of the current treatment options are autograft via mosaicplasty, along with physiotherapy. Another regenerative therapy is BMAC (bone marrow aspirate concentrate), which would have high potential to decrease the rehabilitation time after mosaicplasty [4,5].

### 1.1. Mosaicplasty

Mosaicplasty involves harvesting and transferring a cylindrical osteochondral plug graft from non-weight-bearing areas of the femoral condyles and transplanting it to the recipient region with the chondral or osteochondral defect. Combining different graft sizes will allow the coverage of approximately 80% of the defect, and the free areas between the osteochondral cylinders will be filled with fibrocartilage [5]. 

The International Cartilage Repair Society (ICRS) classification describes the defect’s area, depth, and location [6]. Plugs can be obtained from the medial or lateral peripheral margins of the femoral condyles at the patellofemoral joint. The plugs are transplanted and inserted via fixation and pressing, loaded with healthy cartilage along a converging plane into the newly created sockets in the destination area of equal depth and size. Each stage, harvesting, drilling, and insertion is repeated until the entire defect region has been covered [5,6,7].

The single-use Autograft OATS^®^ (osteochondral autograft transfer system) (Arthrex GmbH, Munich, Germany) is a procedure that facilitates the harvesting of 6 to 12 mm osteochondral cartilage cylinders from the superior and lateral femoral condyle using an autograft donor (>3 cm^2^) [8,9].

### 1.2. BMAC Augmentation

BMAC augmentation is designed to process a mixture of autologous whole blood and bone marrow aspirate. The aspirated marrow obtained from the iliac crest is a source of mesenchymal stem cells, progenitor cells, and growth factors [4]. Neubauer et al. stated that BMAC has the potential to differentiate into various connective tissue cell types, particularly chondrocytes and osteocytes. BMACs have various regulatory features including coordinating functions of regenerative differentiation processes in host cells and immunomodulatory and antiapoptotic functions [10].

Holton et al. stated that BMAC holds promise as a therapeutic approach for chondral injuries, supported by preclinical and clinical studies. In animal models, BMAC enhances defect filling and collagen production [11]. Clinical trials demonstrate macroscopically normal lesions and improved functional outcomes, with BMAC applied alone or with scaffolds like the hyaluronan matrix and collagen [12,13].

### 1.3. Rehabilitation 

Rehabilitation is the final act, and it is crucial to restore and reintegrate the patient into social and professional life. After the completion of the surgical procedure, the immediate goals of rehabilitation include protecting the tissue from shear forces to allow graft incorporation.

Pietschmann et al. emphasized the lack of standardized guidelines for rehabilitation following autologous chondrocyte implantation in the knee and hip, with their survey of tissue regeneration clinical trial group members resulting in the formulation of a rehabilitation protocol. After knee retropatellar surgery, patients are instructed to engage in full extension, undertake continuous passive motion (CPM) for 3–8 h per day starting 24 h post-surgery for 6 weeks, maintain touch-down contact for 6 weeks with gradual weight increases of 15–20 kg per week, initiate sports such as swimming and biking (without resistance) after 6 weeks, and incorporate low-impact activities for 3 months while gradually reintroducing high-impact activities over a period exceeding 12 months. Knee femoral surgeries involve no restrictions on the range of motion (ROM) and follow a rehabilitation protocol similar to knee retropatellar procedures [14].

Edwards et al. highlighted that autologous chondrocyte implantation (ACI) is now firmly established for treating full-thickness chondral defects in the knee, with matrix-induced ACI (MACI) being the latest advancement. Their proposed seven-stage post-operative rehabilitation protocol for tibiofemoral MACI surgery aims to address patient progress and graft maturation, structured into phases based on physical function and tissue healing. The initial phase, occurring within the first 7 days post-surgery, emphasizes pain management, joint mobility restoration, and muscle tone improvement, while minimizing stress on the implant site to prevent complications, employing cryotherapy, a controlled range of motion exercises, and partial weight-bearing education as crucial components [15].

Rehabilitation made in a targeted manner and at the optimal time will enhance the immediate and long-term benefits of mosaicplasty. Through using the BMAC technique as a means of accelerating recovery after mosaicplasty, a quality autograft will be obtained which will then integrate very easily. 

The study aimed to demonstrate that patients who received BMAC augmentation exhibited better outcomes than those who did not. We also aimed to show that patients who followed physiotherapy rehabilitation protocols for 12 weeks had better outcomes than those who followed physiotherapy rehabilitation protocols for just 6 weeks.

## 2. Materials and Methods

### 2.1. Study Design

A single-center randomized controlled trial (RCT) study was conducted between January 2021 and July 2023 in the orthopedics and physiotherapy departments of the Micromedica Clinic in Piatra Neamt and the Emergency County Hospital Piatra Neamt, Orthopedics Department. The study was approved by the Ethics Committee for Scientific Research of Micromedica Clinics in Piatra Neamț with no. 28 from 21 January 2021 and was conducted by the Helsinki Declaration of Ethical Principles. All patients included in the study signed informed consent forms. 

The study was conducted on a group of 37 physically average patients. Criteria for inclusion in the study were as follows: symptomatic chondral or osteochondral defects less than 3 cm in the weight-bearing region of the femoral condyle that do not have elevated inflammatory markers above the accepted values (normal hemoleucograme, sedimentation rate of haematites, CRP); age < 50 years; diagnosis confirmed with imagistic techniques; no glucocorticoids or viscoelastic infiltrations administered in the last 12 months; and patients who have not had synovitis of the knee in the last 12 months. 

Exclusion criteria from the study were as follows: age > 50 years; cartilage defects greater than 3 cm; inability to retrieve donor plug from the non-weight bearing area; and other diseases that are contraindicated for intervention (knee osteoarthritis, osteoporosis, obesity, metabolic diseases, acute rheumatic diseases, ligament lesions, lower limb disuse or less than 120° ROM of flexion, and moderate hydrarthrosis).

The patients were randomly divided into three groups. All the participants were diagnosed with cartilage defects based on a magnetic resonance imaging (MRI) scan of the knee. 

Group 1—(*n* = 10) mosaicplasty + 12-week two-phase rehabilitation and BMAC augmentation; 

Group 2—(*n* = 15) mosaicplasty + 12-week two-phase rehabilitation; 

Group 3—(*n* = 12) mosaicplasty + 6 weeks one-phase rehabilitation. 

### 2.2. Surgical Treatment and BMAC Augmentation

**Figure 1:**
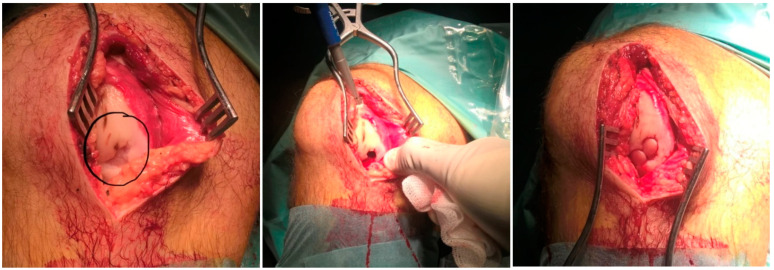
Mosaicplasty procedure using the Artherx OATS^®^ technique. In the left photo, we see the chondral lesion, in the middle photo, the plug extraction, and in the right photo, the plugs are loaded with intact cartilage.

**Figure 2:**
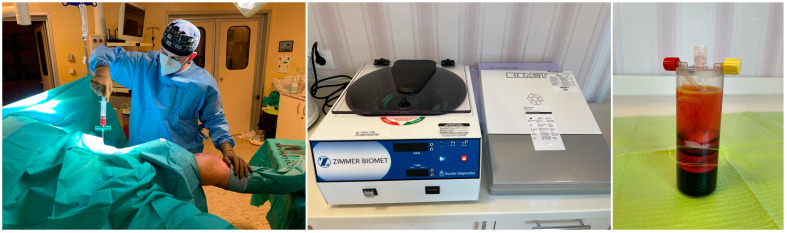
Bone marrow aspirate concentrate (BMAC) augmentation using the BioCUE^®^ BMAC—Zimmer Biomet^®^ kit. The bone marrow was extracted from the anterior superior iliac spine (ASIS), selected for its accessibility during arthroscopy, as illustrated in the left image by the orthopedist. The harvested material is then processed using the Druker/Biologics 755VES Biomet Centrifuge depicted in the middle, followed by additional concentration using the BioCUE^®^ Blood and Bone Marrow Aspirate (BBMA) Concentration System shown on the right side.

The surgery will be performed at Piatra Neamt Emergency County Hospital, radio-imaging investigations will be performed both in the radiology and imaging department of the Hospital and at the Micromedica Clinic, with 1.5 Tesla MRIs. Mosaicplasty was performed with Arthrex OATS^®^ disposable kit (Arthrex GmbH, Munich, Germany) (Figure 1), and BMAC augmentation was performed with BioCUE^®^ BMAC—Zimmer Biomet^®^ kit (Zimmer Biomet, Warsaw, IN, USA) (Figure 2).

After the initial arthroscopic examination, the size and location of the chondral defect are assessed after appropriate debridement. Graft harvesting was then performed from the periphery of the non-weight-bearing trochlea. To prepare for graft insertion at the recipient site, a drill guide was inserted perpendicular to the joint surface into the defect to allow for graft socket reaming. Grafts were spaced at approximately 3 mm to avoid tunnel confluence and the loosening of condyles. It is crucial for the orientation and depth of the graft insertion to mimic the native curvature of the affected joint surface. 

Following mosaicplasty, BMAC augmentation was performed for Group 1. The bone marrow was aspirated from the anterior superior iliac spine (ASIS) due to the patient’s position during arthroscopy—lying on their back with the knee flexed at 90 degrees and supported with a special platform. The aspiration technique involved a single needle insertion with 2–3 redirections, drawing approximately 15–20 mL of marrow with each redirection. The aspiration syringe had a capacity of 60 mL. The bone marrow aspirate was then processed using the Druker/Biologics 755VES Biomet Centrifuge. Initially, the aspirate underwent centrifugation at 2400 rpm for 10 min to remove platelet-poor plasma (PPP), followed by a second centrifugation at 3400 rpm for 6 min. After the second centrifugation, the top layer of the concentrate was introduced into the joint, potentially being guided by ultrasound.

After 72 h, each patient started a physiotherapy program for 6 and 12 weeks, respectively.

**Figure 3:**
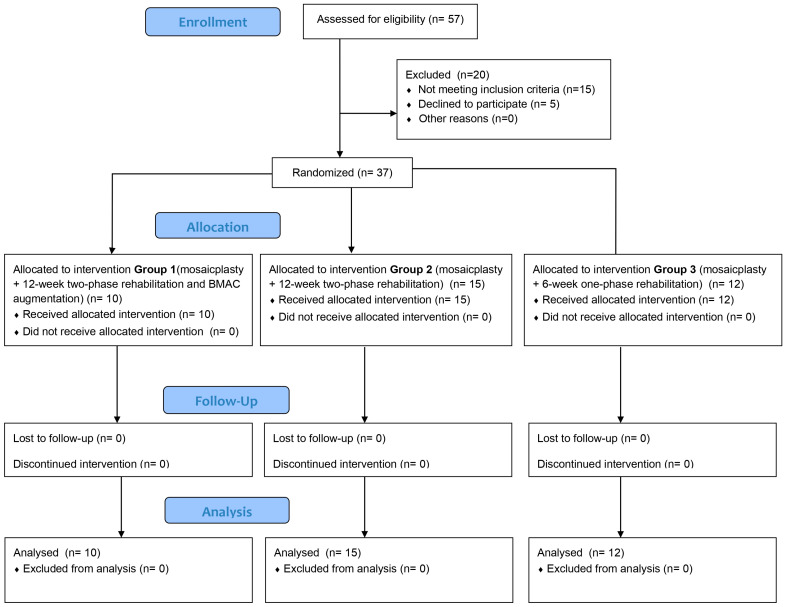
CONSORT flow diagram.

The rehabilitation of the subjects was carried out in the Micromedica Medical Centre in the physiotherapy department, with physiotherapists trained in post-surgery knee rehabilitation. The study was conducted according to the Consolidated Standards of Reporting Trials (CONSORT) model (Figure 3).

### 2.3. Subjects Evaluation

The orthopedic physician clinically evaluated the subjects and confirmed the presence of chondral lesions. The visual analog scale (VAS) was used to assess post-surgery pain initially at 6 weeks and again at 12 weeks. Goniometry was used to initially assess the knee ROM 6 and 12 weeks post-surgery. Clinical tests included manual muscle testing (MMT) for the evaluation of the quadricep force, which was made initially 6 and 12 weeks post-surgery. The Western Ontario and McMaster University Arthritis Index (WOMAC) was used to evaluate the pain, stiffness, and function of the knee. The questionnaire consists of 24 questions, which the patients self-complete, and which characterize the severity of pain and functional deficit, taken initially 6 and 12 weeks post-surgery.

### 2.4. Physiotherapy Treatment (PT)

Phase 1 (weeks 0–6 postoperatively) aims to relieve pain, restore full passive flexion extension, and regain quadricep control. To control pain, inflammation, and edema, we used conventional TENS for 40 min, using two channels and a frequency of 100 Hz and 100 μs pulse (IONOSON-Expert PHYSIOMED ELEKTROMEDIZIN AG, Schnaittach-Laipersdorf, Germany); ultrasound for 8 min of 0.2–0.3 W/cm^2^ at 1 MHz with a duty-cycle of 10%, IONOSON-Expert (PHYSIOMED ELEKTROMEDIZIN AG Schnaittach-Laipersdorf, Germany); 15 min deep oscillation for 10 min at 170–250 Hz and for 5 min at 70–90 Hz, with glove application (Deep Oscillation—PHYSIOMED ELEKTROMEDIZIN AG Schnaittach-Laipersdorf, Germany); and 15–20 min of cryotherapy at a minimum of 4 °C (CRYOPUSH Cold Compression Therapy System, Chengdu, Sichuan, China).

The patient walks with crutches and will wear orthotics which are adjustable in the range of 0–30°. During the first 2 weeks, Artromot CPM is recommended for the passive recovery of the flexion and extension ROM, or passive mobilizations performed by a physiotherapist. After the third week, mobilization on stationary bikes without resistance can be started. Active exercises and isometric contractions should be performed to strengthen the quadriceps. Bearing weight is forbidden, and care should be taken when using orthoses and CPM.

Phase 2 (weeks 6–12) is aimed at improving the lower limb function by progressively increasing muscle strength and endurance. The orthosis is removed, and the lower limb is progressively loaded to the limit of tolerance. Fifteen minutes of low-frequency electrostimulation (EMS) + fifteen minutes of medium-frequency sinusoidal Kotz + isometric contractions are recommended for strengthening the thigh muscles. 

Progressive resistance with ankle weights, partial squats, and back-to-wall slides at 0–30° and a stationary bike with a full ROM on flexion. Ladders or impact exercises and pivoting in VAR or VALG are prohibited, and extension exercises with the full loading of the operated lower limb are also prohibited.

## 3. Results

### 3.1. Statistical Analysis

This study aimed to demonstrate that patients who received BMAC augmentation displayed better outcomes than those who did not. Also, we aimed to demonstrate that patients who followed physiotherapy rehabilitation protocols for 12 weeks demonstrated better outcomes than those who followed physiotherapy rehabilitation protocols for just 6 weeks.

The data were analyzed using ISB SPSS Statistics, V. 25. The differences between the groups were evaluated using the one-way ANOVA test. 

### 3.2. Analysis of Results between Groups for the WOMAC Scale

**Scheme 1:**
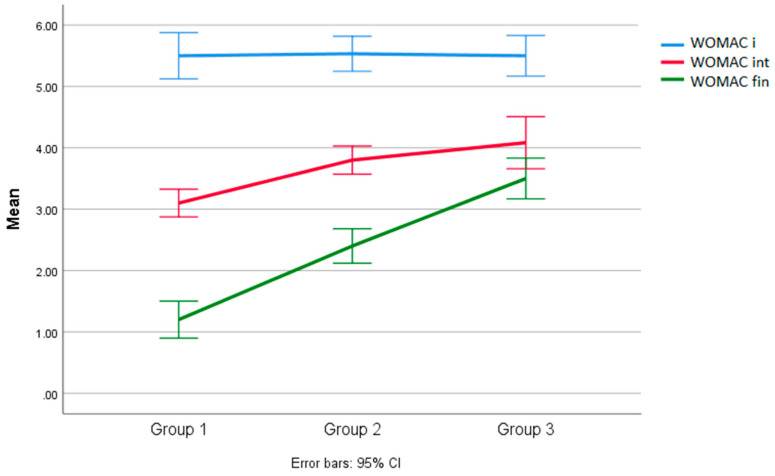
Graphical representation of the evolution of the mean values for the initial (red), intermediate (green), and final (blue) WOMAC scale for Group 1—(*n* = 10) mosaicplasty + 12-week two-phase rehabilitation and BMAC, Group 2—(*n* = 15) mosaicplasty + rehabilitation in two 12-week phases, and Group 3—(*n* = 12) mosaicplasty + single phase rehabilitation for 6 weeks only.

We applied the one-way ANOVA analysis of variance method to test whether there were significant differences in the WOMAC scale scores according to the group to which the subjects were assigned. The results obtained are shown in Table 1 and Scheme 1. 

The initial results of the WOMAC scale do not indicate a statistically significant difference according to the group variable [F (2, 34) = 2.06, *p* < 0.142].

The intermediate results of the WOMAC scale indicate a statistically significant difference according to the group variable [F (2, 34) = 5.24, *p* < 0.010]. To check between which of the three groups the differences are significant, we applied the post hoc t Turkey test. The results indicate significant differences between the subjects in Groups 1 and 3 (Turkey t = 6.23, *p* < 0.015) and between the subjects in Groups 3 and 2 (Turkey t = 5.00, *p* < 0.034), respectively.

The final results of the WOMAC scale indicate a statistically significant difference according to the group variable [F (2, 34) = 111, *p* < 0.000]. To check between which of the three groups the differences are significant, we applied the post hoc t Turkey test. The results indicate significant differences between the subjects in Group 1 and 2 (Turkey t = 3.90, *p* < 0.010), between the subjects in Group 1 and 3 (Turkey t = 17.98, *p* < 0.000), and between the subjects in Group 3 and 2 (Turkey t = 14.08, *p* < 0.000), respectively.

### 3.3. Analysis of between-Group Results for the VAS Scale

**Scheme 2:**
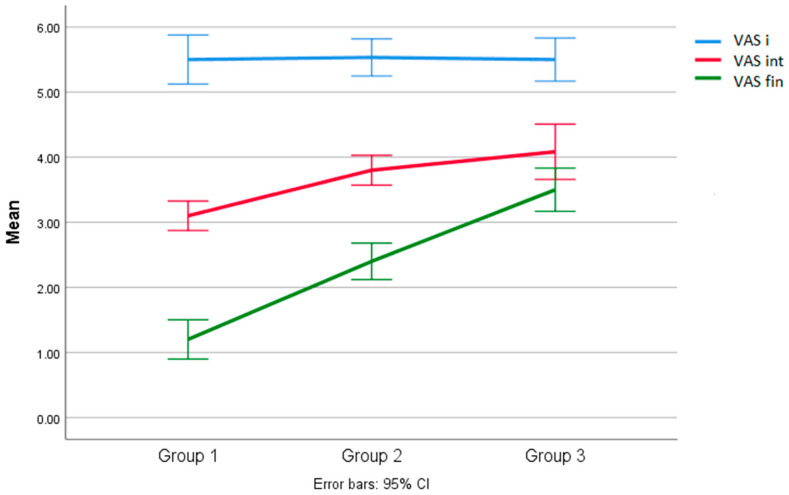
Graphical representation of the evolution of the mean values for the initial (red), intermediate (green), and final (blue) VAS scale for Group 1—(*n* = 10) mosaicplasty + 12-week two-phase rehabilitation and BMAC, Group 2—(*n* = 15) mosaicplasty + rehabilitation in two 12-week phases, and Group 3—(*n* = 12) mosaicplasty + single phase rehabilitation of 6 weeks only.

We applied the one-way ANOVA analysis of variance method to test whether there were significant differences in the VAS scale scores depending on the group to which the subjects were assigned. The results obtained are shown in Table 2 and Scheme 2. 

The initial VAS scale results did not indicate a statistically significant difference according to the group variable [F (2, 34) = 0.18, *p* < 0.982].

The intermediate results of the VAS scale indicate a statistically significant difference according to the group variable [F (2, 34) = 11.40, *p* < 0.000]. To check between which of the three groups the differences are significant, we applied the post hoc t Turkey test. The results indicate significant differences between the subjects in Groups 1 and 2 (Turkey t = 0.70, *p* < 0.004) and between the subjects in Groups 1 and 3 (Turkey t = 0.98, *p* < 0.000), respectively.

The final VAS results indicate a statistically significant difference according to the group variable [F (2, 34) = 59.87, *p* < 0.000]. To check between which of the three groups the differences are significant, we applied the post hoc t Turkey test. The results indicate significant differences between the subjects in Groups 1 and 2 (Turkey t = 1.20, *p* < 0.000), between the subjects in Groups 1 and 3 (Turkey t = 2.30, *p* < 0.000), and between the subjects in Groups 3 and 2 (Turkey t = 1.10, *p* < 0.000), respectively.

### 3.4. Analysis of Results between Groups for the MMT of the Quadriceps

**Scheme 3:**
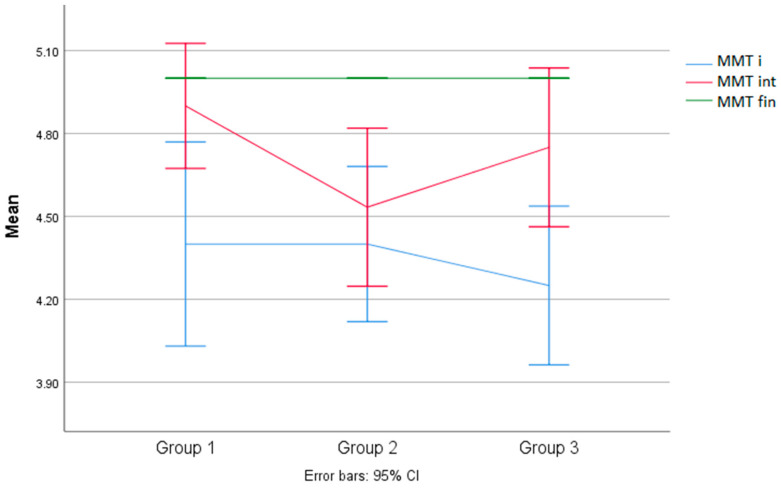
Graphical representation of the evolution of the mean values for the initial (red), intermediate (green), and final (blue) MMT quadricep force (Grade 5) for Group 1—(*n* = 10) mosaicplasty + 12-week two-phase rehabilitation and BMAC, Group 2—(*n* = 15) mosaicplasty + rehabilitation in two 12-week phases, and Group 3—(*n* = 12) mosaicplasty + single phase rehabilitation of 6 weeks only.

We applied the one-way ANOVA analysis of variance method to test for significant differences in the MMT scores (quadricep force) depending on the group to which the subjects were assigned. The results obtained are shown in Table 3 and Scheme 3. 

The initial strength results did not indicate a statistically significant difference according to the group variable [F (2, 34) = 0.376, *p* < 0.689].

The intermediate MMT results indicate no statistically significant difference by group variable [F (2, 34) = 2.090, *p* < 0.139]. 

The final MMT results could not be compared using the one-way ANOVA test because all subjects achieved a maximum level of quadricep force (Grade 5).

### 3.5. Analysis of the between-Group Results of the Knee ROM Values

**Scheme 4:**
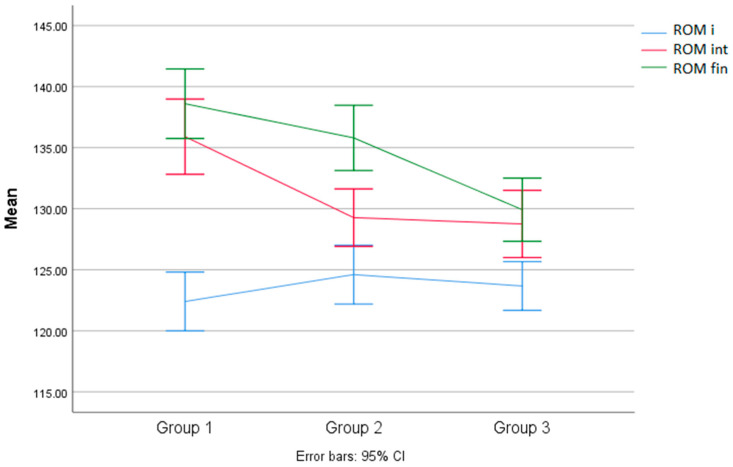
Graphical representation of the evolution of the mean values for the initial (red), intermediate (green), and final (blue) knee ROM scale for Group 1—(*n* = 10) mosaicplasty + 12-week two-phase rehabilitation and BMAC, Group 2—(*n* = 15) mosaicplasty + rehabilitation in two 12-week phases, and Group 3—(*n* = 12) mosaicplasty + single phase rehabilitation of 6 weeks only.

To test whether there were significant differences in the knee ROM results according to the group to which the subjects were assigned, we applied the one-way ANOVA analysis of variance method. The results obtained are shown in Table 4 and Scheme 4. 

The initial ROM results indicate no statistically significant difference according to the group variable [F (2, 34) = 1.037, *p* < 0.366].

The intermediate ROM results indicate a statistically significant difference according to the group variable [F (2, 34) = 9.38, *p* < 0.001]. To test between which of the three groups the differences are significant, we applied the post hoc t Turkey test. The results indicate significant differences between the subjects in Groups 1 and 2 (Turkey t = 6.63, *p* < 0.002) and between the subjects in Groups 1 and 3 (Turkey t = 7.15, *p* < 0.001), respectively.

The final knee ROM results indicate a statistically significant difference according to the group variable [F (2, 34) = 11.60, *p* < 0.000]. To test between which of the three groups the differences are significant, we applied the post hoc t Turkey test. The results indicate significant differences between the subjects in Groups 1 and 3 (Turkey t = 8.68, *p* < 0.000) and between the subjects in Groups 2 and 3 (Turkey t = 5.83, *p* < 0.004).

## 4. Discussion

Based on our study findings, it appears that patients who underwent mosaicplasty and adhered to a 12-week rehabilitation protocol with BMAC augmentation (Group 1) or without BMAC augmentation (Group 2) experienced better outcomes when compared to those who received mosaicplasty without BMAC augmentation and underwent only a 6-week rehabilitation protocol (Group 3). Specifically, patients in Group 1 showed superior results in terms of pain reduction, as evidenced by the lower WOMAC index scores. Additionally, patients who completed the full 12-week rehabilitation program displayed better outcomes when compared to those who only completed a 6-week program, indicating the effectiveness of the extended rehabilitation duration. These findings highlight the potential benefits of BMAC augmentation and adherence to a comprehensive rehabilitation protocol in improving outcomes following mosaicplasty procedures.

According to the manufacturer’s data, the efficiency of the BMAC processing in yielding PRP was evaluated based on recovery rates and concentration factors. Nucleated cell recovery from the aspirate was found to be 77.5%, indicating successful concentration into the PRP product. Similarly, platelet recovery stood at 71%, demonstrating effective extraction and concentration during processing. The resulting PRP showed a substantial increase in the platelet count with a concentration factor of 7.2x when compared to the original aspirate. The nucleated cell concentration in the PRP was also notably higher with a factor of 7.9x. These findings underscore the method’s efficiency in producing a concentrated PRP rich in nucleated cells, which holds promise for enhancing tissue repair and regenerative therapy [16].

BMAC has shown promise as a therapeutic option for symptomatic knee osteoarthritis (OA). Research studies have indicated that BMAC injections can effectively enhance pain relief and patient-reported outcomes in knee OA patients, with significant improvements being demonstrated when compared to other treatments like platelet-rich plasma (PRP) and microfragmented adipose tissue [17,18]. 

Skowroński et al. demonstrated the efficacy of one-stage reconstruction using BMAC and collagen membranes for large cartilage lesions. With 52 out of 54 patients experiencing significant improvements across all assessment scales and no reported complications, the findings underscore BMAC therapy as a reliable and effective treatment option. Notably, the observed average improvements of 25 points in the Knee Injury and Osteoarthritis Outcome Score (KOOS) and 35 points in the Lysholm scales one year post-surgery highlight the substantial functional benefits conferred by this approach. Moreover, the study’s assertion of BMAC’s cost-effectiveness compared to autologous chondrocyte transplantation further strengthens its appeal in clinical practice [19]. 

According to Krych et al., in a comparative study, patients undergoing scaffold implantation augmented with BMAC demonstrated superior cartilage maturation compared to those treated with PRP or scaffold alone. Twelve months post-surgery, BMAC-treated groups exhibited greater cartilage fill and mean T2 values closer to those of superficial native hyaline cartilage using MRI. These findings suggest that BMAC augmentation may enhance early cartilage repair and maturation [20].

Wang et al. concluded that, despite the theoretical benefits, the addition of BMAC did not lead to significant improvements in osseous integration at the graft–host bony interface following osteochondral allograft transplantation. Despite the theoretical benefits of BMAC in enhancing graft incorporation, our findings suggest that its use did not lead to observable changes in bone, cartilage, or ancillary features when compared to osteochondral allograft transplantation alone. While BMAC remains a promising therapeutic option, its efficacy in enhancing osseous integration in osteochondral allograft transplantation procedures requires careful consideration and additional investigation [21].

The findings from Solheim et al. suggest that patients undergoing OATS in either the patellofemoral joint or the medial/lateral femoral condyles can expect similar long-term outcomes. Survival analysis revealed no significant differences in the distribution of survival between the two groups, indicating the comparable effectiveness of OATS regardless of the lesion location within the knee joint. Despite nearly half of the cases experiencing eventual failure within 18 years, many patients retained reasonably good knee function for an extended period post-OATS, irrespective of whether the lesion was in the patellofemoral or femoral condyles. These results emphasize the durability and effectiveness of OATS as a treatment option for cartilage repair in various regions of the knee joint [22].

In an original study concerning 60 knee arthroscopic cases conducted by Zhou et al., the VAS and WOMAC scores were applied before surgery and 6 and 12 weeks and then 6 and 12 months after the surgery of two groups of patients. The experimental group was treated with cell concentrates containing mesenchymal stromal cells. Differences in the VAS and WOMAC scores show that the short-term results of the study indicate that knee arthroscopy with mesenchymal stromal cell concentrates is effective, reduces pain, and improves function in patients with chondral knee injuries [23].

While there may not be specific studies focusing solely on the application of transcutaneous electrical nerve stimulation (TENS) in mosaicplasty procedures, TENS has been utilized as a complementary modality for pain management in various orthopedic contexts, including postoperative care following mosaicplasty surgeries.

TENS are rectangular single-phase or biphasic currents that selectively activate large-diameter non-nociceptive afferents (A-β) to reduce nociceptor sensitivity and activity at a segmental level [2,24]. 

Zhou et al. demonstrated that electrical stimulation has potential regulatory factors in increasing extracellular matrix synthesis, proliferation, and differentiation, and, in vitro, can be considered as a tissue engineering method to improve cartilage repair [25].

The application of TENS currents reduces levels of pro-inflammatory cytokines in the blood post-treatment sessions, thereby controlling pain through a completely different mechanism than originally described [26,27].

Clinical studies have demonstrated the physiological effects of Deep Oscillation^®^, showcasing its efficacy in reducing pain, exerting anti-inflammatory effects, and facilitating the reabsorption of edema. This innovative therapy has been shown to alleviate pain through its ability to modulate pain perception and promote the relaxation of muscles and tissues. Additionally, Deep Oscillation^®^ (PHYSIOMED ELEKTROMEDIZIN AG Schnaittach-Laipersdorf, Germany) exhibits anti-inflammatory properties via promoting lymphatic drainage and reducing local inflammation [28]. Moreover, its oscillatory action aids in the reabsorption of edema by enhancing fluid movement within tissues, thereby reducing swelling and promoting tissue healing. Overall, these findings underscore the therapeutic potential of Deep Oscillation^®^ in managing various musculoskeletal conditions and promoting tissue recovery [29].

Vladeva et al. have shown that Deep Oscillation^®^ is an effective method for increasing the ROM and reducing pain, inflammation, and swelling in the early rehabilitation of patients after knee joint arthroplasty [30].

Ultrasound (US) is a widely used form of physiotherapy for treating musculoskeletal conditions due to its non-invasive nature and safety profile. By utilizing high-frequency sound waves, US therapy penetrates deep into tissues, promoting various therapeutic effects [2,31,32]. The US not only relieves symptoms, but can also provide joint cartilage repair effects. Several studies have shown that the US stimulates collagen formation, regulates inflammatory responses, and induces cartilage repair [2,33]. Priscila Daniele de Oliveira Perrucini et al. showed that low-intensity pulsed US had a bio-stimulatory effect on fibroblast cells in vitro at 0.2 W/cm^2^ with a 10% duty cycle. After 48 h US, IL-6 cytokine production and gene modulation were affected, confirming its therapeutic properties on tissue healing [34].

Cryotherapy is a post-operative intervention that is safe, inexpensive, and easy to administer, often used in the control of inflammation, edema, and pain management [35]. In an original study, van Ooij et al. demonstrated that cryotherapy after total knee arthroplasty provides faster rehabilitation and improved short-term ROM knee flexion [36].

In this study, we introduced cryotherapy at the end of the physiotherapy program to reduce the knee temperature, which can rise during physiotherapy exercises to protect the donor plug, and to control edema and pain during physiotherapy sessions.

Physiotherapy exercises are used to limit the loss of joint function and increase the muscle strength, lower limb stability, and coordination. The main benefits of physiotherapy exercises are improved physical function and quality of life. For exercise to be effective in controlling chronic inflammation, a minimum of 150 min per week of moderate-intensity exercise or at least 75 min per week of vigorous-intensity exercise is required [37,38].

The protocol used by the orthopedic department at the Massachusetts General Hospital includes several rehabilitation steps. Before surgery, the orthopedic surgeon recommends the use of a stationary bike for two weeks to return your knee ROM as close to normal as possible. After surgery, it is recommended CPM, starting from 0–50° and gradually increasing to 100°, as well as quadricep setting, heel propping, heel slides, sitting hell slides, and ankle pumps [39].

Inderhaug et al. recommend that, after the first non-weight-bearing phase of rehabilitation, the program should focus on the ROM gain of the knee, with a gradual increase in neuromuscular exercises. Patients are usually advised to postpone returning to sports until 6 months after surgery at the earliest [40].

Healthy knee joint function relies on intact cartilage, making the accurate assessment of cartilage health crucial. According to Nischal et al., MRI stands out as the most dependable tool for evaluating chondral lesions. Additionally, MRI plays a vital role in monitoring progress following the surgical management of cartilage defects and in assessing post-surgical outcomes [41]. The modified Outerbridge classification is used to assess the cartilage defects of traumatic etiology based on MRI in a clinical scenario, based on the arthroscopic assessment of the knee. The classification is on four grades, of which grade zero corresponds to normal articular cartilage appearance and grade IV corresponds to full-thickness chondral defects and exposed subchondral bone. The Outerbridge classification correlates well with the arthroscopic chondral defect score suggested by the International Cartilage Repair Society (ICRS) to help surgical planning, and also has prognostic implications [42,43,44].

This study contained some limitations as only one disposable mosaicplasty kit was used for all patients regardless of age, gender, and weight in Groups 1, 2, and 3. Another limitation of this study was the use of a single PT protocol of 6–12 weeks; future studies should focus on treatment protocols with longer periods in different combinations with new technologies such as TECAR radiofrequency, the Super Inductive System, etc. Another important limitation was the small number of patients evaluated and the evaluation interval, which was only 12 weeks. Since changes were observed in the clinical characteristics of subjects in Group 1, future studies will be conducted with a larger number of BMAC-augmented subjects and more clinical evaluation and imaging intervals in order to unlock the potential of this study.

## 5. Conclusions

This study explored various regenerative treatment methods and rehabilitation approaches aimed at managing symptomatic chondral defects in the weight-bearing area of the knee. The results offer valuable insights for refining treatment strategies and enhancing patient outcomes in this complex clinical situation.

Firstly, our results highlight the importance of incorporating biological augmentation, such as BMAC, into surgical interventions like mosaicplasty for chondral defect repair. BMAC, with its rich source of mesenchymal stem cells and growth factors, has shown promising potential in enhancing tissue regeneration and promoting cartilage repair. The significant improvements observed in pain relief, knee function, and ROM among patients receiving BMAC augmentation underscore its role as a valuable adjunct to surgical interventions for chondral defects.

Secondly, the duration and intensity of postoperative rehabilitation play a crucial role in determining treatment outcomes. Our study demonstrated that patients who underwent a longer rehabilitation program (12 weeks) achieved superior functional outcomes when compared to those who completed a shorter protocol (6 weeks). This suggests that extended rehabilitation may be necessary to optimize the benefits of surgical interventions and facilitate the restoration of normal joint biomechanics and function. 

Furthermore, our findings emphasize the importance of a multidisciplinary approach to managing chondral defects, involving close collaboration between orthopedic surgeons, rehabilitation specialists, and other healthcare professionals. A comprehensive treatment strategy that integrates surgical techniques, biological augmentation, and tailored rehabilitation protocols can address the complex nature of chondral defects and improve patient satisfaction and quality of life.

## Figures and Tables

**Table 1:** One-way ANOVA test results for the WOMAC scale.

Initial Assessment Results								
Group	M	SD	DF		F	Sig.	Turkey t	
1	64.1	2.92	InterG	IntraG	2.06	<0.142	Mean Difference	Sig.
2	64.86	4.24	2	34			-	-
3	66.91	2.53						
Interim evaluation results							Turkey t	
Group	M	SD	DF		F	Sig.	Mean Difference	Sig.
1	30.10	6.80	InterG	IntraG	5.24	<0.010	G1/G2 = 1.23	<0.813
2	31.33	4.67	2	34			G1/G3 = 6.23	<0.015
3	36.33	3.02					G3/G2 = 5.00	<0.034
Final evaluation results							Turkey t	
Group	M	SD	DF		F	Sig.	Mean Difference	Sig.
1	15.10	3.07	InterG	IntraG	111	<0.000	G1/G2 = 3.90	<0.010
2	19.00	2.53	2	34			G1/G3 = 17.98	<0.000
3	33.08	3.60					G3/G2 = 14.08	<0.000

Legend: M = mean, SD = standard deviation, DF = degrees of freedom, InterG = intergroups, IntraG = intragroups, G1 = group 1, G2 = group 2, G3 = group 3.

**Table 2:** One-way ANOVA test results for the VAS scale.

Initial Assessment Results								
Group	M	SD	DF		F	Sig.	Turkey t	
1	5.50	0.52	InterG	IntraG	0.18	<0.982	Mean Difference	Sig.
2	5.53	0.51	2	34			-	-
3	5.50	0.52						
Interim evaluation results							Turkey t	
Group	M	SD	DF		F	Sig.	Mean Difference	Sig.
1	3.10	0.31	InterG	IntraG	11.40	<0.000	G1/G2 = 0.70	<0.004
2	3.80	0.41	2	34			G1/G3 = 0.98	<0.000
3	4.08	0.66					G3/G2 = 0.28	<0.309
Final evaluation results							Turkey t	
Group	M	SD	DF		F	Sig.	Mean Difference	Sig.
1	1.20	0.42	InterG	IntraG	59.87	<0.000	G1/G2 = 1.20	<0.000
2	2.40	0.50	2	34			G1/G3 = 2.30	<0.000
3	3.50	0.52					G3/G2 = 1.10	<0.000

Legend: M = mean, SD = standard deviation, DF = degrees of freedom, InterG = intergroups, IntraG = intragroups, G1 = group 1, G2 = group 2, G3 = group 3.

**Table 3:** One-way ANOVA test results for the MMT of the quadriceps.

Initial Assessment Results								
Group	M	SD	DF		F	Sig.	Turkey t	
1	4.40	0.51	InterG	IntraG	0.376	<0.689	Mean Difference	Sig.
2	4.40	0.50	2	34			-	-
3	4.25	0.45						
Interim evaluation results							Turkey t	
Group	M	SD	DF		F	Sig.	Mean Difference	Sig.
1	4.90	0.31	InterG	IntraG	2.090	<0.139	-	-
2	4.53	0.51	2	34				
3	4.75	0.45						
Final evaluation results							Turkey t	
Group	M	SD	DF		F	Sig.	Mean Difference	Sig.
1	5.0000	0.00	InterG	IntraG	-	-	-	-
2	5.0000	0.00	2	34				
3	5.0000	0.00						

Legend: M = mean, SD = standard deviation, DF = degrees of freedom, InterG = intergroups, IntraG = intragroups, G1 = group 1, G2 = group 2, G3 = group 3.

**Table 4:** One-way ANOVA test results for the knee ROM.

Initial Assessment Results								
Group	M	SD	DF		F	Sig.	Turkey t	
1	122.40	3.37	InterG	IntraG	1.037	<0.366	Mean Difference	Sig.
2	124.60	4.35	2	34			-	-
3	123.66	3.14						
Interim evaluation results							Turkey t	
Group	M	SD	DF		F	Sig.	Mean Difference	Sig.
1	135.90	4.30	InterG	IntraG	9.38	<0.001	G1/G2 = 6.63	<0.002
2	129.26	4.25	2	34			G1/G3 = 7.15	<0.001
3	128.75	4.33					G3/G2 = 1.66	<0.984
Final evaluation results							Turkey t	
Group	M	SD	DF		F	Sig.	Mean Difference	Sig.
1	138.60	3.97	InterG	IntraG	11.60	<0.000	G1/G2 = 2.80	<0.274
2	135.80	4.82	2	34			G1/G3 = 8.68	<0.000
3	129.91	4.07					G3/G2 = 5.83	<0.004

Legend: M = mean, SD = standard deviation, DF = degrees of freedom, InterG = intergroups, IntraG = intragroups, G1 = group 1, G2 = group 2, G3 = group 3.

## Data Availability

Data are contained within the main text of the article.
